# Validation of gene regulatory network inference based on controllability

**DOI:** 10.3389/fgene.2013.00272

**Published:** 2013-12-12

**Authors:** Xiaoning Qian, Edward R. Dougherty

**Affiliations:** ^1^Department of Electrical and Computer Engineering, Texas A & M UniversityTX, USA; ^2^Computational Biology Division, Translational Genomics Research InstitutePhoenix, AZ, USA

**Keywords:** network inference, genetic regulatory network, control, validation, probabilistic Boolean network

## Abstract

There are two distinct issues regarding network validation: (1) Does an inferred network provide good predictions relative to experimental data? (2) Does a network inference algorithm applied within a certain network model framework yield networks that are accurate relative to some criterion of goodness? The first issue concerns scientific validation and the second concerns algorithm validation. In this paper we consider inferential validation relative to controllability; that is, if an inference procedure is applied to data generated from a gene regulatory network and an intervention procedure is designed on the inferred network, how well does it perform on the true network? The reasoning behind such a criterion is that, if our purpose is to use gene regulatory networks to design therapeutic intervention strategies, then we are not concerned with network fidelity, *per se*, but only with our ability to design effective interventions based on the inferred network. We will consider the problem from the perspectives of stationary control, which involves designing a control policy to be applied over time based on the current state of the network, with the decision procedure itself being time independent. The objective of a control policy is to optimally reduce the total steady-state probability mass of the undesirable states (phenotypes), which is equivalent to optimally increasing the total steady-state mass of the desirable states. Based on this criterion we compare several proposed network inference procedures. We will see that inference procedure ψ may perform poorer than inference procedure ξ relative to inferring the full network structure but perform better than ξ relative to controllability. Hence, when one is aiming at a specific application, it may be wise to use an objective-based measure of inference validity.

## 1. Introduction

Network validity can be approached from two perspectives: *scientific* and *inferential*. Scientific validity is an epistemological issue concerning the ability of a network model to yield observations concordant with those predicted by the model (Dougherty and Bittner, 2011). It involves relations between model characteristics and experimental observations such that mathematical predictions based on the model are manifested in the phenomena via these relations. Inferential validity concerns the ability of an inference procedure to operate on data generated from the model and yield an inferred model close to the original network relative to some distance function. Inferential validity is purely a mathematical issue concerning the inference algorithm. The two issues, scientific and inferential validity, are not unrelated because in practice an inferential procedure is used to construct a model from real data and the scientific validity is therefore dependent upon the performance of the inferential procedure. In this paper we are interested in inferential validity [see Dougherty (2011) for a discussion of the two types of validity].

The validity of inference procedures for gene regulatory networks is discussed in Dougherty (2007), where validation is relative to some network characteristic and quantified by some distance between the characteristic for the original network and the characteristic for the inferred network, such as a norm between the steady-state distributions of the original and inferred networks. Generally speaking (we shall be more rigorous shortly), (1) a characteristic is derived for the network; (2) a data sample is generated from the network; (3) an inference procedure operates on the sample to produce an inferred network; (4) the corresponding characteristic is derived for the inferred network; (5) the corresponding characteristics for the original and inferred networks are compared; and (6) the validity of the inference procedure is determined by some distance between the characteristics.

The preceding validation protocol focuses solely on the network itself, not any objective to which the network is to be used, although clearly successful use of the inferred network will depend to some extent on the closeness of the inferred and original networks. Our aim here is to characterize the notion of *objective inferential validity*, where inferential validity is measured relative to the objective for which the network will be used. In particular, we are concerned with controllability. Specifically, if the objective is to derive a control procedure from the inferred network, then it is of utmost importance that the control procedure works well on the original network (from which the sample data have been generated). In other words, to what extent is controllability preserved by the inference procedure? It may be that the original and inferred networks are a quire discordant; however, if their lack of agreement has little impact on derivation of the control procedure, then this lack of agreement is of little consequence.

**Network Inference**

- Q: What types of biological networks have been inferred in the paper?
- A: We focus on the inferential validity of genetic regulatory network inference. We evaluate and compare different inference algorithms in the framework of probabilistic Boolean networks (PBNs) by both synthetic random PBNs and a melanoma metastatic network inferred from gene expression data.
- Q: How was the quality/utility of the inferred networks assessed?
- A: We propose and discuss different inferential validity criteria for inferring genetic regulatory networks, including (1) Hamming distance to measure the network topology closeness; (2) steady-state mass difference for network dynamic behavior similarity; and (3) expected difference of desirable steady-state mass shift by applying derived optimal control when the operational objective is intervention. We would like to emphasize that objective inferential validity criteria based on operational objectives such as intervention are viable choices when we typically do not have the ground truth of real-world gene regulatory networks.
- Q: How were these networks validated?
- A: Both synthetic random networks and the melanoma metastatic network are considered as benchmark networks. From these network models, we simulate the network dynamics with perturbations, inferred networks by different algorithms are evaluated by ground truth network models based on the aforementioned three inferential validity criteria.

Two basic intervention approaches have been considered for gene regulatory networks in the framework of probabilistic Boolean networks (PBNs) (Dougherty and Datta, 2005; Datta and Dougherty, 2007; Shmulevich and Dougherty, 2007), structural intervention and external control. Both take advantage of the fact that the probabilistic characteristics of a PBN are characterized by an associated Markov chain. *Structural intervention* involves a one-time change of the network structure (wiring) to beneficially alter the long-run behavior (steady state) of the network (Shmulevich et al., 2002b; Xiao and Dougherty, 2007; Qian and Dougherty, 2008). Given a class of potential structural changes, the problem is to find the optimal structural intervention resulting in a desired alteration of the steady-state distribution. *Stationary control* is generally based on flipping (or not flipping) the value of a control gene(s) over time in an effort to favorably move the steady-state mass. Efforts have mainly focused on infinite-horizon stationary external control. The first proposed approach utilizes dynamic programming to find an optimal policy relative to a cost function, in which case the steady-state distribution is altered as a by-product of this optimization (Pal et al., 2006). A second approach is to utilize a greedy (no optimality) algorithm to find a policy that directly aims at altering the steady-state distribution Qian et al. (2009). Here we will use a more recently proposed approach for gene regulatory networks that uses linear programming to find a policy that is optimal relative to minimizing undesirable steady-state mass (Yousefi and Dougherty, 2013). This latter approach avoids the introduction of a subjectively defined cost function as in Pal et al. (2006) and avoids the sub-optimality of greedy algorithms (Qian et al., 2009). Instead, the amount of shift in the steady-state distribution gives an intrinsic network measure, as it also does in the case of structural intervention. The situation is analogous to classification, where the Bayes error is intrinsic to the feature-label distribution, as opposed to errors resulting from suboptimal classifiers that have been derived from data via some *ad hoc* classification rule. In this paper we restrict our attention to stationary control because it is very possible that the optimal structural controller for an inferred network is based on an inferred function that may not exist in the original network. In such a case it would not be feasible to apply the identified intervention for the inferred network back to the original network.

**Figure 1:**
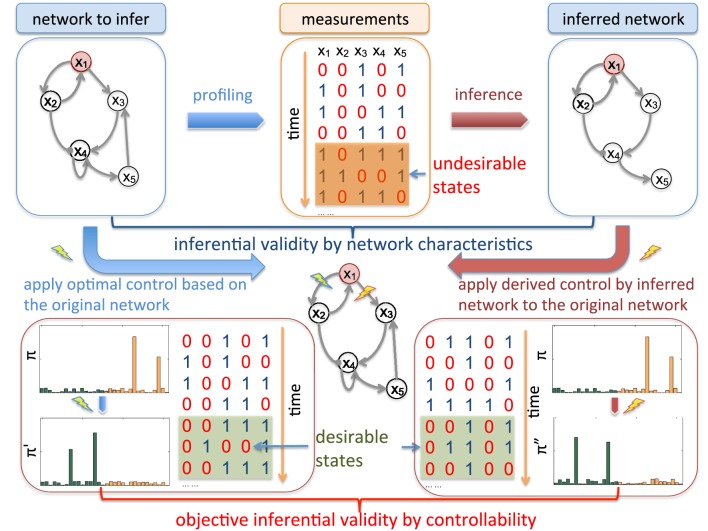
**Schematic illustration of inferential validity**. There are different criteria to evaluate inferred networks from available temporal measurements. For example, we can directly measure the difference of inferred regulatory relationships among genes by the commonly adopted Hamming distance between the original network adjacency matrix and the inferred adjacency matrix. We are interested in objective-based inferential validity based on controllability. For example, assuming that *x*_1_ is a genetic marker marked in red, the network is considered in “undesirable” states when it is up-regulated (*x*_1_ = 1). Hence, from the translational perspective, the ultimate goal of studying this network system is to develop effective therapeutic strategies based on collected data from the system. Hence when evaluating network inference algorithms, instead of comparing other network characteristics, it may be more appropriate to directly investigate how the derived intervention strategies based on inferred networks perform on the original networks by reducing the long-run probability of entering into undesirable states, which leads to our controllability-based inferential validity. As shown in the figure, assume that we derive the optimal control based on the original network to block the regulation from *x*_1_ to *x*_2_ while the derived control from the inferred network is to block the regulation from *x*_1_ to *x*_3_. Note that both of the derived control policies have to be validated on the true network. One criterion to evaluate the inferred network as our “objective-based inferential validity” is to check how the steady-state distribution π″ by blocking *x*_1_ → *x*_3_ on the original network compares to the optimally controlled steady-state distribution π^'^ after blocking *x*_1_ → *x*_2_ with respect to the reduction of undesirable steady-state mass in the original steady-state distribution π before intervention. This difference reflects the cost of using the derived control from the inferred network instead of the optimal control designed from the true network.

Figure 1 illustrates the main idea of objective inferential validity for quantifying the performance of different network inference procedures with respect to controllability. Assuming that we are interested in an impaired biological system that has a higher risk of entering into aberrant phenotypes, from the collected measurements, our goal is to design effective stationary control policies to reduce the risk of entering into these undesirable or bad states. One way to characterize network states is based on the prior knowledge of biomarkers. As a hypothetic example, *x*_1_ in Figure 1 is considered as the marker gene, whose value being 1 (up-regulated) are not desirable as it may represent metastasizing phenotypes in cancerous systems, for example. Based on what we can observe, from microarray profiling or other high-throughput techniques, we may infer the underlying network model that governs the state dynamics. Many previous inferential validity measures are solely interested in the network itself. However, in this scenario, inference procedures should be evaluated in regard to our final objective of effectively reducing the undesirable risk by evaluating the control performance of intervention strategies derived using the network model inferred from partially observed data. In fact, in real-world scenario, we typically do not have the ground truth of the underlying system. Objective inferential validity may be the only reasonable framework for network inference validation.

## 2. Systems and methods

### 2.1. Probabilistic boolean networks

Probabilistic Boolean networks (Shmulevich et al., 2002a) extend the classical Boolean networks (Kauffman, 1969, 1993) by introducing uncertainty in the rule structure [see Shmulevich and Dougherty (2010) for a comprehensive review]. This uncertainty is motivated by randomness in the inference procedure, inherent biological randomness, and model stochasticity owing to latent variables outside the model that are involved in regulation.

A binary Boolean network *G*(*V, F*) is defined by a set *V* = {*x*_1_, *x*_2_ …, ,*x*_*n*_} of binary variables, *x*_*i*_ ∈ {0, 1}, *i* = 1, …, *n*, and a list of Boolean functions *F* = (*f*_1_, *f*_2_, …, *f*_*n*_). The value of *x*_*i*_ at time *t* + 1 is completely determined by a subset {*x*_*i*1_, *x*_*i*2_, …, *x*_*ik*_*i*__} ⊂ *V* at time *t* via a Boolean function *f*_*i*_: {0, 1}^*k*_*i*_^ → {0, 1}. Transitions are homogeneous in time and we have the update *x*_*i*_ (*t* + 1) = *f*_*i*_(*x*_*i*1_(*t*), *x*_*i*2_(*t*), …, *x*_*ik*_*i*__ (*t*)). Each *x*_*i*_ represents the state (expression) of gene *i*, where *x*_*i*_ = 1 and *x*_*i*_ = 0 represent gene *i* being expressed and not expressed, respectively. It is commonplace to refer to *x*_*i*_ as the *i*th gene. The list *F* of Boolean functions represents the rules of regulatory interactions between genes. All genes are assumed to update synchronously in accordance with the functions assigned to them and this process is then repeated. At any time *t*, the state of the network is defined by a state vector **x**(*t*) = (*x*_1_(*t*), *x*_2_(*t*), …, *x*_*n*_(*t*)), called a *gene activity profile* (GAP). Given an initial state, a BN will eventually reach a set of states, called an *attractor cycle*, through which it will cycle endlessly. Each initial state corresponds to a unique attractor cycle and the set of states leading to a specific attractor cycle is known as the *basin of attraction* (BOA) of the attractor cycle.

A *Boolean network with perturbation* (BNp) is defined by allowing each gene to possess the possibility of randomly flipping its value with a positive probability *p*. Implicitly, we assume that there is an i.i.d. random perturbation vector γ = (γ_1_, γ_2_, …, γ_*n*_), where γ_*i*_ ∈ {0, 1}, the *i*th gene flips if and only if γ_*i*_ = 1, and *p* = *P* (γ_*i*_ = 1) for *i* = 1, 2, …, *n*. If **x**(*t*) is the GAP at time *t*, then the next state **x** (*t* + 1) is either **f**(**x**(*t*)) with probability (1 − *p*)^*n*^ or **x**(*t*) ⊕ γ with probability 1 − (1 − *p*)^*n*^, where **f** is the multi-output function from the truth table and ⊕ is component-wise addition modulo 2. Larger values of *p* result in the regulatory rules being overridden by random alterations to the regulatory signaling, which one might call “noise.”

A binary *probabilistic Boolean network* (PBN) is composed of a family {*B*_1_, *B*_2_, …, *B*_*m*_} of BNps together with probabilities governing the selection of a BNp at each time. The *m* constituent BNps are characterized by *m* network functions, {**f**_1_, **f**_2_, …, **f**_*m*_}. At any time point there is a positive probability *q* of switching from the current governing constituent BNp (context) to another, with the selection probabilities for transitioning to *B*_1_, *B*_2_, …, *B*_*m*_ given by *c*_1_, *c*_2_, …, *c*_*m*_, respectively. Note that the probability of switching to any constituent network *B*_ℓ_, 1 ≤ ℓ ≤ *m* is independent of the current network; indeed, when a switch is called for, the current network may “switch” to itself. By definition, a PBN inherits the attractor cycles of its constituent BNps. There are two modeling interpretations regarding *q*. If *q* < 1, the PBN is said to be *context-sensitive* (Brun et al., 2005); if *q* = 1, as in the original formulation of PBNs (Shmulevich et al., 2002a), then the PBN is said to be *instantaneously random*. The modeling interpretation is that there are latent variables outside the network model controlling the context of the network and larger values of *q* correspond to greater effects of latent variables. Although we have defined PBNs as having binary gene values, there is nothing inherent in this restriction and the general definition assumes that each gene can take a finite number of values, say in the set {0, 1, …, *d*}.

Transition rules of any PBN can be modeled by a homogeneous Markov chain, whose states of the transition probability matrix (TPM) *p* are the GAPs of the PBN [see Faryabi et al. (2009) for the particulars on how the Markov chain is derived for different classes of PBNs]. Perturbation makes the corresponding Markov chain of a PBN irreducible and ergodic. Hence, the network possesses a steady-state distribution π^*T*^ = π^*T*^ *P*, describing its long-run behavior. For small *q* and *p*, most of the steady-state mass lies in the attractors of the PBN (Brun et al., 2005), which by definition are the attractors of the constituent BNs. Let 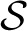 = {(**x**, y) : **x** ∈ 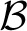, *y* ∈ {1, 2, …, *m*}} be the state space of the PBN, where 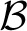 denotes the space of all GAPs or network states for any constituent BN with *n* genes and *y* is the index to which constituent BN currently governs the dynamics. We note that when we have BNps with only one constituent BN, *y* is redundant. Let {**Z**_*k*_ ∈ 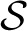, *k* = 0, 1,…} be the stochastic process of the state of the PBN that has both the information about the current constituent BN and GAP of the underlying network. Originating from state **i** ∈ 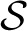, the successor state **j** ∈ 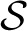 is selected randomly according to the TPM *p*, with its **ij**th element defined by *p*_**ij**_ ≜ *P*(***Z***_*k* + 1_ = **j** |***Z***_*k*_ = **i**) for all *k* = 0, 1, ….

### 2.2. Maximal steady-state alteration

we now briefly outline the setting in which an infinite-horizon policy can be found that achieves maximal steady-state alteration, meaning that it optimizes the shift of steady-state mass from undesirable to desirable states. let 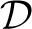 and 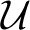 denote the sets of desirable and undesirable states, respectively. one way to define 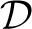 and 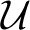 is based on the values of given genetic markers as illustrated in Figure 1. for instance, undesirable states may be those in which gene wnt5a is up-regulated because such states are associated with increased risk of metastasis in melanoma, whereas the desirable states would be those in which wnt5a is down-regulated (see section 4.3). we assume that the pbn admits an external control input *A* from a set of actions, 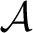, specifying the type of intervention on a set of control genes. for instance, *A* = 0 may indicate no-intervention and *A* = 1 may indicate that the expression level of a single gene, *g*^*c*^, *c* ∈ {1, 2, …, *n*}, is flipped. in this intervention scenario, the control action *A* = 1 at state (**x**, *y*) replaces the row corresponding to the state (**x**, *y*) in the original tpm of the underlying markov chain by the row corresponding to the state (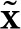, *y*), where the binary representation of 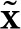 is the same as **x** except in bit *v*^*c*^, where it is flipped.

Denote by {**z**_*k*_, *k* = 0, 1, …} and {*a*_*k*_, *k* = 0, 1, …} the sequences of observed states and actions. A *policy* is a prescription for taking actions at each time point *k*. Actions may be taken in accordance with a random mechanism, possibly a function of the entire history of the system up to time *k*. For time *k*, let *h*_*k*_ = (**z**_0_, *a*_0_, **z**_1_, *a*_1_, …, **z**_*k*_, *a*_*k*_) denote the observed history. A policy υ = (υ_0_, υ_1_, …) is a sequence prescribed by the decision maker that steers the dynamics of the underlying system. If the history *h*_*k* − 1_ is observed up to time *k*, then the decision maker chooses an action *a* ∈ 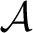
(**z**_*k*_) with probability υ_*k*_(*a* | *h*_*k* − 1_, **z**_*k*_).

The goal is to find an intervention policy to maximally shift the long-run probability mass of undesirable states to desirable ones. Let 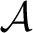 = 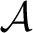
(**j**) = {0, 1} for all **j** ∈ 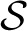. The amount of shift in the aggregated probability of undesirable states for a PBN controlled under υ is defined as 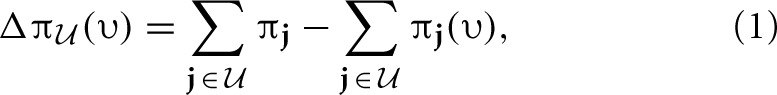 where π and π(υ) are the steady-state vectors for the Markov chains governed by the original and controlled PBNs, respectively. The goal is to maximize Δπ_
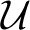
_(υ). An optimal policy that is both stationary (time-invariant) and deterministic can be obtained by solving a linear programming problem, which we refer to as the Maximal Steady-State Alteration (MSSA) algorithm (Yousefi and Dougherty, 2013). The optimal policy depends on the choice of undesirable states and the control input. In our case, these will be determined by the values of certain genes, which can be considered as *a priori* known biomarkers for example. Since we are interested in quantifying the performance of inference procedures on the network, these marker genes will be selected randomly for random networks without loss of generality.

### 2.3. Inferential validation

Network comparison is based on a distance function, μ, which need only be a semi-metric because we do not want to require that μ(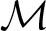, 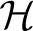) = 0 implies 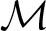 = 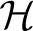, the point being that we compare networks via characteristics and two distinct networks might possess the same characteristic yet be quite different. For instance, consider the steady-state distribution. If π = (π_1_, π_2_, …, π_*m*_) and ω =(ω_1_, ω_2_, …, ω_*m*_) are the steady-state distributions for networks 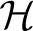 and 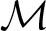, respectively, then a network distance is defined by μ_*ss*_(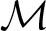, 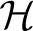) = ∥π − ω∥, where ∥•∥ is some vector norm. As a second example, suppose one is interested in network topology. Define the adjacency matrix in the following manner: given an *n*-gene network, for *i*, *j* = 1, 2, …, *n*, the (*i*, *j*) entry in the matrix is 1 if there is a directed edge from the *i* th to the *j*th gene; otherwise, the (*i*, *j*) entry is 0. If **A** =(*a*_*ij*_) and **B** = (*b*_*ij*_) are the adjacency matrices for networks 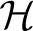 and 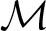, respectively, where 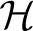 and 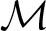 possess the same gene set, then the Hamming distance between the networks is defined by μ_ham_(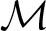, 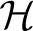) = ∑^*n*^_*i, j* = 1_ |*a*_*ij*_ − *b*_*ij*_|. Both μ_*ss*_ and μ_ham_ are semi-metrics.

Focusing on full network inference (and following Dougherty, 2007), the goodness of an inference procedure ψ relative to distance μ is measured by μ(ψ (*S*), 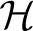), where 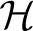 is the original network and sample *S* is a realization of the random process, Σ, governing data generation from 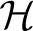. Hence, μ (ψ(Σ), 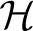) is a random variable and the performance of ψ is characterized by the distribution of μ (ψ(Σ), 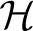), which depends on the distribution of Σ. We adopt the expectation of the distribution of μ(ψ(Σ), 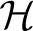) as the measure for inferential validity, *E*_Σ_ [μ(ψ(Σ), 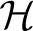)] taken with respect to Σ.

Rather than considering a single network, we can consider a distribution, H, of random networks, where the occurrences of realizations 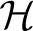 of H are governed by a probability distribution. Averaging over the class of random networks, our interest focuses on *E*_H_ [*E*_Σ_ [μ(ψ(Σ), 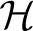)]]. Inference procedure ψ_1_ is better than the inference procedure ψ_2_ relative to the distance μ, the random network H, and the sampling procedure Σ if *E*_H_ [*E*_Σ_ [μ(ψ_1_(Σ), 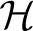)]] < *E*_H_ [*E*_Σ_ [μ(ψ _2_(Σ), 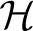)]]. In practice, the expectation must be estimated by an average 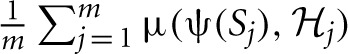, where *S*_1_, *S*_2_, …, *S*_*m*_ are sample point sets generated according to Σ from networks 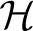
_1_, 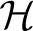
_2_, …, 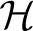
_*m*_ randomly chosen from H.

The preceding analysis applies unchanged when measuring validity relative to controllability; indeed, it is just a matter of defining the distance function. Let 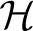 denote the original network, *S* be a sample generated from 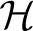, υ_
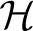
_ and υ_ψ(*S*)_ be the maximal steady-state alteration policies for 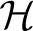 and ψ(*S*), respectively, and π^
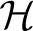
^ and π^ψ(*S*)^ be the steady-state vectors for 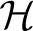 controlled by υ_
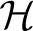
_ and υ_ψ(*S*)_, respectively. Then the inferential-validity distance relative to controllability is defined by 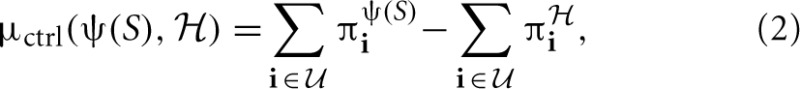 where 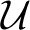 is the class of undesirable states. Applying this distance to a distribution **H**, of random networks yields the expectation in which we are interested, namely, 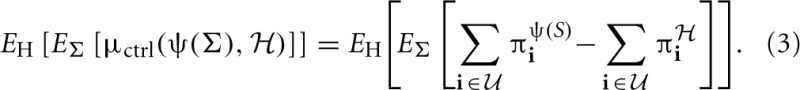


For analyzing PBNs, we are confronted by computational issues in regard to transition probability matrices of their underlying Markov chains. In the case of controlling binary discrete-time networks, one is looking at a matrix of dimension *N* × *N*, where *N* is the number of states. For a PBN, *N* = *m* × 2^*n*^, where *m* is the number of contexts and *n* is the number of genes. Generally speaking, networks beyond 15 genes become computationally intractable with regard to deriving control policies. Larger networks require first the application of a reduction algorithm to reduce the size of the state space (Qian and Dougherty, 2009b; Ivanov et al., 2010; Qian et al., 2010). These inevitably lose information. If one is going to study inference for networks larger than 15 genes, then the analysis must include the reduction algorithm as part of the design. This can certainly be done but it would not essentially change the kind of inference analysis in which we are involved. The price would be that, whereas by using the MSSA algorithm the entire matter is intrinsic, there being no subjective cost functions, prior use of a reduction algorithm would destroy the intrinsic nature of the analysis.

### 2.4. Network inference algoritms

Learning regulatory relationships among genes is a major challenge in computational biology. Numerous methods based on different mathematical models have been developed; however, performance evaluation remains problematic (Marbach et al., 2010). In this paper, we focus on network inference algorithms for PBNs from one or several time series of observed gene expression states **x**(*t*). We have implemented a few commonly adopted inference algorithms for PBNs with modifications to allow for more than one time series: REVEAL (REVerse Engineering ALgorithm) and its extension (Liang et al., 1998; Akutsu et al., 1999; Murphy and Mian, 1999; Martin et al., 2007), MDL (Minimal Description Length) (Tabus and Astola, 2001; Zhao et al., 2006; Dougherty et al., 2008), and Best-Fit (Lähdesmäki et al., 2003; Marshall et al., 2007; Lähdesmäki and Shmulevich, 2012).

These inference algorithms aim for identifying regulatory relationships among genes as well as finding corresponding Boolean functions for them so that the observed state transitions in time series data are most “consistently” explained by the inferred functions. For example, REVEAL (Liang et al., 1998) identifies predictors for each gene by estimating the mutual information between the temporal profile of each gene and all the combination profiles of potential genes as regulators, starting from one regulator per gene. In order to find a unique solution, in the worst case, the algorithm requires an exponential number of state transitions in the observed time course data, with respect to the number of genes *n* in the network. However, as most of biological networks are sparse (Arnone and Davidson, 1997; Thieffry et al., 1998), REVEAL works effectively in practice and (Akutsu et al., 1999) also have proven that only *O*(log *n*) state transitions are required when the maximum number of predictors, *K* = max^*n*^_*i* = 1_ *k*_*i*_, for all the genes in the network is small. However, the original REVEAL algorithm and the exhaustive algorithm in Akutsu et al. (1999) focus on inferring BNs instead of PBNs and require finding the “consistent” Boolean functions for each gene. They assume that the observed time course data themselves are completely consistent based on underlying Boolean functions without errors.

With random perturbations introduced in PBNs, instead of finding consistent Boolean functions, the inference algorithm Best-Fit (Lähdesmäki et al., 2003; Marshall et al., 2007; Lähdesmäki and Shmulevich, 2012) searches for the best-fit function for each gene by exhaustively searching for all the combination of potential regulator sets. Similarly, with small *K*, the algorithm is feasible with a given number of state transitions and is efficient with the time complexity *O*(*m*log *m*poly(*n*)) with *m* state transitions, in which poly(*n*) is time to compute the minimum error for one given state transition Lähdesmäki et al. (2003). For our implementations (Murphy and Mian, 1999; Lähdesmäki et al., 2003; Lähdesmäki and Shmulevich, 2012) based on both REVEAL and Best-Fit algorithms, we have modified the algorithms to get both regulator sets and corresponding best-fit functions. Finally, with a limited number of observed state transitions and potential random perturbations, the inferred regulatory functions may still be *partially defined Boolean functions* (Lähdesmäki et al., 2003). To obtain a unique solution, we can further impose other biologically motivating constraints. For example, in Pal et al. (2005), BNs are inferred simply based on the attractor structure of network dynamics, which can be extended to impose dynamic constraints to search for suitable solutions.

In this work, we adopt the MDL-based network inference algorithm (Tabus and Astola, 2001; Zhao et al., 2006; Dougherty et al., 2008) to penalize the model complexity of inferred networks. We have modified the algorithm proposed in Zhao et al. (2006) to identify the best regulator set with the minimum combination of network coding length, capturing the model complexity, and data coding length, which is similar to REVEAL based on mutual information. The MDL network coding length in Zhao et al. (2006) has similar asymptotic performance to the Bayesian Information Criterion (BIC) model complexity, which we also have implemented in our set of inference algorithms. Finally, both MDL (Zhao et al., 2006) and BIC (Murphy and Mian, 1999) adopt *ad hoc* measures of model description length that necessitate tuning parameters as weighting coefficients to balance the model and data coding lengths (Tabus and Astola, 2001; Dougherty et al., 2008) and inference performances or validity measures may change with different tuning parameters. To overcome this difficulty, we also adopt a universal MDL (uMDL) network inference algorithm (Dougherty et al., 2008) in which the model and data coding length together is a theoretical measure derived from a universal normalized maximum likelihood model and no tuning parameters are needed (Tabus and Astola, 2001).

## 3. Implementation

We will compare network inference algorithms for their inferential validity based on both synthetic networks as well as a well-studied metastatic melanoma network (Bittner et al., 2000; Kim et al., 2002; Weeraratna et al., 2002; Qian and Dougherty, 2008; Yousefi and Dougherty, 2013).

To evaluate the inference algorithms based on simulated time series of network states, we first generate random PBNs with properties that resemble those of biological networks so that we have the ground truth networks for validation. For appropriate evaluation, we have imposed a few assumptions: First, as genetic regulatory networks are commonly believed to have sparse connectivity topology, we have restricted the Boolean functions in random PBNs to have at most five predictors: *K* = max^*n*^_*i* = 1_ *k*_*i*_ ≤ 5. This assumption also enables all the inference algorithms to run smoothly on these random PBNs as the computational complexity of these algorithms, especially those based on exhaustive enumerations, reduces significantly as shown in Akutsu et al. (1999); Lähdesmäki et al. (2003). Second, as the network state space is exponential with respect to the number of genes or the network size, the number of state transitions observed will usually not be large enough to uniquely determine the network structure and thereafter the regulatory functions. For the inference algorithms adopted in this paper, all of which are based on solving the consistency problem (Liang et al., 1998; Akutsu et al., 1999; Lähdesmäki et al., 2003; Zhao et al., 2006; Martin et al., 2007), we take the most sparse network as the final solution within the feasible networks that give the same minimum prediction errors in REVEAL and Best-Fit or the same objective function values in the inference algorithms with BIC and MDL regularization. The motivation is that biological networks are usually stable and robust to random perturbations and larger *k*_*i*_ leads to increased sensitivity of the steady-state distribution to random gene perturbations Shmulevich and Dougherty (2007), Qian and Dougherty (2009a, 2010).

With either simulated or real ground truth networks, we can generate time series of gene expression profiles with different numbers of state transitions based on their underlying Markov chains so that we can investigate the inference performances with different available sample sizes. We have implemented REVEAL, MDL, BIC, uMDL, and Best-Fit to infer networks with these simulated time series. Our implementations of these different algorithms are based on the PBN Toolbox (http://code.google.com/p/pbn-matlab-toolbox/), the Bayes Net Toolbox (https://code.google.com/p/bnt/), as well as the source code provided by the authors of Dougherty et al. (2008). The detailed descriptions of these different algorithms can be found in the corresponding papers (Liang et al., 1998; Murphy and Mian, 1999; Lähdesmäki et al., 2003; Zhao et al., 2006; Dougherty et al., 2008; Lähdesmäki and Shmulevich, 2012).

We compute three distance functions μ(ψ(*S*), 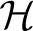) to evaluate an inference algorithm ψ: (1) the Hamming distance μ_ham_; (2) the *L*_1_ norm μ_*ss*_ between the steady-state distributions of ψ(*S*) and 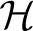; and (3) the controllability distance μ_ctrl_ defined in (2). For inferential validity based on controllability, we find the optimal stationary control policies for the original and inferred networks based on the MSSA algorithm (Yousefi and Dougherty, 2013).

## 4. Results and discussion

### 4.1. Simulated BNps with 7 genes

We first evaluate different inference algorithms on synthetically generated random networks. We generate 1000 random BNps with *n* = 7 genes, maximum input degree *K* = 3, and perturbation probability *p* = 0.01. For each node, we uniformly assign 1 to *K* regulators. Hence the average connectivity in this set of random networks is 2. After determining the regulatory relationships among nodes, the regulatory functions for each node are determined by randomly filling in the corresponding truth tables with Bernoulli random numbers with the bias following a Beta distribution with mean 0.5 and standard deviation 0.01. For each random BNp, we simulate time series of different numbers of state transitions based on its underlying Markov chain. The number of “observed” state transitions *m* ranges from 10 to 60 to reflect the difficulty level of network inference. For control, we choose the first node as the marker gene and define the undesirable states as these network states with the first node down-regulated. In the binary representation of network states, 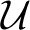 = {**x**|*x*_1_ = 0}. As the networks are randomly generated, without loss of generality, we allow intervention on the last node as the control gene, which we can either knock up or down to derive control policies. In our simulated random BNps, we have the original average undesirable steady state mass π^org^_
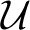
_ = 0.5071 with standard deviation 0.3575, with π^org^_
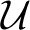
_ ≈ 0.5 because we set the bias to 0.5. When we apply the MSSA algorithm to derive the optimal stationary control policies for these random BNps, the average controlled undesirable steady state mass is π_
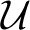
_ = 0.3703 with the standard deviation 0.3749.

Based on these simulated time series, we have implemented REVEAL, BIC, MDL, uMDL, and Best-Fit inference algorithms and modified accordingly to reconstruct BNps, including regulatory relationships and regulatory functions represented as general truth tables. For BIC and MDL, we set the regularization coefficients to values previously reported to have good performance in Zhao et al. (2006), λ = 0.5 for BIC and λ = 0.3 for MDL.

Table 1 provides the network inferential validity measurements: normalized Hamming distance μ_ham_ (Hamming distance over the total number of edges in true networks), the steady-state distance μ_*ss*_, and the controllability distance μ_ctrl_ for different network inference algorithms given different numbers of state transitions. As discussed in (Zhao et al., 2006), BIC and MDL perform similarly. Regarding the accurate recovery of regulatory relationships, it is interesting to see that Best-Fit appears to achieve the best performance with respect to μ_ham_ while REVEAL does not perform very well. One explanation could be that REVEAL introduces many false positives, hopefully to best fit the data by using the functions with more regulators. This is in fact what we observe from our experiments. All the other inference algorithms choose the functions with the smallest number of regulators either by complexity regularization in BIC, MDL, and uMDL; or choosing the “parsimonious” functions with the minimum prediction errors in Best-Fit. For uMDL, we note that μ_ham_ improves quickly with the increasing sample size compared to other complexity regularization algorithms BIC and MDL. Based on our experiments, uMDL consistently generates very low false positive edges (close to zero), even with a very limited number of samples, which is the main advantage of the uMDL algorithms. This has also been shown in the original paper (Dougherty et al., 2008). For μ_*ss*_, both REVEAL and Best-Fit perform consistently better than BIC, MDL, and uMDL, since both REVEAL and Best-Fit aim to find the network models that best fit the observed state transitions. With regularization on model complexity by BIC, MDL, and uMDL, the steady-state distances are greater. As mentioned earlier, REVEAL and Best-Fit, especially REVEAL, reconstruct networks with more edges to explain the observed data, which leads to smaller μ_*ss*_.

**Table 1:** **The comparison of network inference algorithms (REVEAL, BIC, MDL, uMDL, and Best-Fit) with *M* different number of observed state transitions**.

**Validity**	**μ_ham_**			**μ_*ss*_**			**μ_ctrl_**		
**M**	**10**	**30**	**50**	**10**	**30**	**50**	**10**	**30**	**50**
REVEAL	0.7774	0.6111	0.6511	0.6743	0.4657	0.4216	0.1067	0.0275	0.0049
BIC	0.6966	0.4196	0.3304	0.8679	0.7089	0.5492	0.0739	0.0300	0.0126
MDL	0.7204	0.4260	0.3294	0.9414	0.7225	0.5435	0.0775	0.0311	0.0121
uMDL	0.8000	0.3728	0.2471	1.1957	0.6973	0.4935	0.1058	0.0352	0.0093
Best-Fit	0.7311	0.3919	0.2913	0.6378	0.4244	0.4098	0.1027	0.0250	0.0045

When we investigate the inferential validity with respect to controllability, μ_ctrl_, we see interesting changes of tendency between the five algorithms. Especially with very few state transitions, *M* = 10, BIC, MDL, and uMDL algorithms perform better than REVEAL and Best-Fit, which indicates that the regularization on model complexity with a limited number of observations helps reconstruct network models that yield better controllers. With more observations, REVEAL and Best-Fit gradually perform better than BIC, MDL, and uMDL due to introduced bias by model complexity regularization.

**Figure 2:**
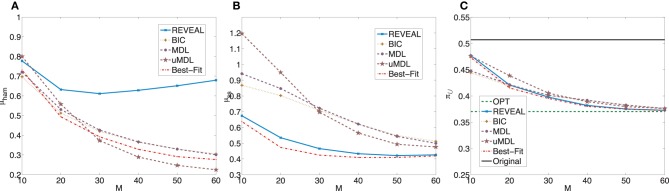
**Performance comparison of five network inference algorithms by different validity indices based on simulated BNps with 7 genes and *K* = 3. (A)** Average normalized Hamming distance μ_ham_; **(B)** μ_*ss*_; **(C)** average undesirable steady-state mass π_
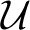
_ after applying derived stationary control policies based on inferred networks to the original ground truth BNps, compared to the average undesirable mass obtained by the optimal control policy (OPT) based on the complete knowledge of original BNps and the average undesirable mass before intervention (Original).

Figure 2 plots μ_ham_, μ_*ss*_, and the average undesirable steady-state mass using the control policy designed on the inferred network via the MSSA algorithm. For comparison purposes, the latter average is compared to the average original undesirable mass and the average undesirable mass following application of the MMSA control policy designed on the original network. As *m* increases from 10 to 60, all algorithms improve. In fact, with more than 50 observed state transitions for these generated random BNps, the derived stationary control policies achieve almost the same performance compared to the optimal control policies with complete knowledge of the network models. The average performances from inferred networks are in fact within 5% for all five inference algorithms when *M* = 60.

**Figure 3:**
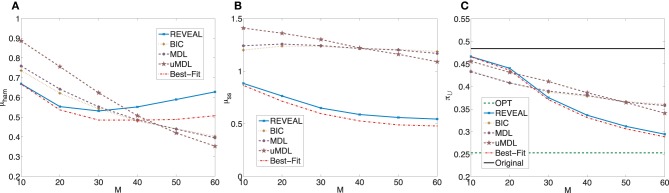
**Performance comparison of five network inference algorithms by different average validity indices based on BNps with 7 genes and *K* = 5. (A)** Average normalized Hamming distance μ_ham_; **(B)** μ_*ss*_; **(C)** average undesirable steady-state mass π_
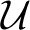
_ after applying derived stationary control policies based on inferred networks to the original ground truth BNps, compared to the average undesirable mass obtained by the optimal control policy (OPT) based on the complete knowledge of original BNps and the average undesirable mass before intervention (Original).

We further evaluate inference algorithms on a similar set of 1000 random BNps with *n* = 7 genes with the same settings but change the maximum input degree *K* = 5, which increases the average connectivity to 3. For this set of random BNps, we have the average undesirable original steady state mass π^org^_
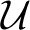
_ = 0.4841 with standard deviation 0.3171. When we apply the MSSA algorithm to derive the optimal stationary control policies for these random BNps, the average controlled undesirable steady state mass is π_
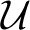
_ = 0.2529 with the standard deviation 0.3144. The average shift of undesirable masses is higher compared to the previous set of random networks, which is expected as the network sensitivity monotonically increases with the average network connectivity (Kauffman, 1993; Shmulevich and Dougherty, 2007; Qian and Dougherty, 2009a). With higher sensitivity, networks can be more effectively controlled. We again compare the inferential validity as in the previous experiment. Figure 3 shows plots analogous to Figure 2. Especially, we note that in this set of experiments, we can achieve close-to-optimal intervention with fairly small sample size as illustrated in Figure 3C. It is clear that the performance of different inference algorithms depends on the characteristics of the networks, especially the network sensitivity. More specifically, all three indices become worse for all the inference algorithms, illustrating that with increasing network sensitivity, the inference problem becomes more difficult. It is also clear that the performance improves at slower rates with the increasing sample size when we have higher network sensitivity. Another important difference is that for this set of random networks, both REVEAL and Best-Fit have higher μ_ham_ when the number of samples increase above 40. The reason may be due to the tendency of random perturbations forcing both algorithms to bias toward more complex Boolean functions with more input variables as regulators.

### 4.2. Simulated BNps with 9 genes

**Figure 4:**
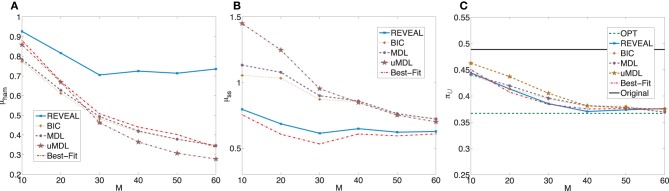
**Performance comparison of five network inference algorithms by different average validity indices based on BNps with 9 genes and *K* = 3**. **(A)** Average normalized Hamming distance μ_ham_; **(B)** μ_*ss*_; **(C)** average undesirable steady-state mass π_
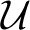
_ after applying derived stationary control policies based on inferred networks to the original ground truth BNps, compared to the average undesirable mass obtained by the optimal control policy (OPT) based on the complete knowledge of original BNps and the average undesirable mass before intervention (Original).

For simulations with 9 genes, owing to run time, we generate 200 BNps with *n* = 9 genes and perturbation probability *p* = 0.01. We again make uniformly random assignments of 1 to *K* regulators, with *K* = 3 so that the average connectivity is 2. The bias for the corresponding truth tables follows the same Beta distribution with mean 0.5 and stand deviation 0.01. The number of “observed” state transitions *m* range from 10 to 60. The derivation of control policies is still based on the definition of the undesirable states 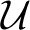 = {**x**|*x*_1_ = 0} and the last node is the control gene. In the simulated random BNps, the average undesirable steady state mass is π^org^_
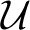
_ = 0.4886 with the standard deviation 0.3764. When we apply the MMSA algorithm to derive the optimal stationary control policies for these random BNps, the average controlled undesirable steady state mass is π_
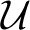
_ = 0.3668 with the standard deviation 0.3863. Figure 4 shows plots analogous to Figure 2 with the trends similar as those observed in the previous experiments with corresponding random BNps with 7 genes and *K* = 3.

**Figure 5:**
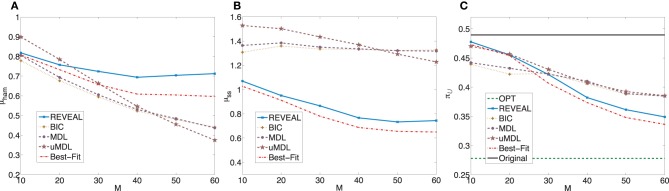
**Performance comparison of five network inference algorithms by different average validity indices based on BNps with 9 genes and *K* = 5**. **(A)** Average normalized Hamming distance μ_ham_; **(B)** μ_*ss*_; **(C)** average undesirable steady-state mass π_
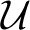
_ after applying derived stationary control policies based on inferred networks to the original ground truth BNps, compared to the average undesirable mass obtained by the optimal control policy (OPT) based on the complete knowledge of original BNps and the average undesirable mass before intervention (Original).

In the second set of simulated random BNps with 9 genes, the settings are the same except that *K* = 5. In these random networks, the average undesirable steady state mass is π^org^_
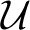
_ = 0.4895 with standard deviation 0.3269. When we apply the MSSA algorithm to derive the optimal stationary control policies for these random BNps, the average controlled undesirable steady state mass is π_
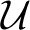
_ = 0.2781 with standard deviation 0.3268. Figure 5 is analogous to Figure 3.

In summary, when we evaluate different inference procedures with respect to different inferential validity criteria, different inference procedures show different trends with their increasing sample size. Their performance overall depends on network characteristics as well as available samples. Finally, when effective intervention is our final operational objectivel, it is promising that we can achieve effective intervention based on inferred networks, even with fairly small sample size as illustrated in Figures 2C, 3C, 4C, 5C.

### 4.3. A metastatic melanoma network

**Figure 6:**
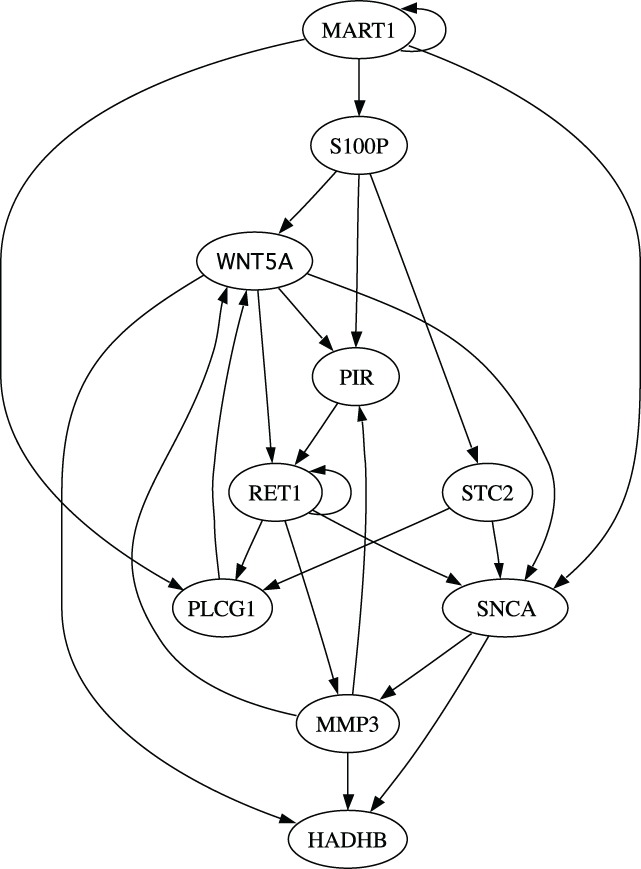
**Multivariate relationships among genes in the metastatic melanoma**.

Finally, we evaluate different inference algorithms based on a metastatic melanoma network used in previous studies on network intervention (Qian and Dougherty, 2008; Qian et al., 2009; Yousefi and Dougherty, 2013). The network has 10 genes listed in the order from the most to the least significant bit: WNT5A, PIR, S100P, RET1, MMP3, PLCG1, MART1, HADHB, SNCA, and STC2. The order does not affect our analysis. We note here that this network was derived from gene expression data (Kim et al., 2002) collected in studies of metastatic melanoma (Bittner et al., 2000; Weeraratna et al., 2002). Table 2 and Figure 6 together illustrate the regulatory relationships among these selected 10 genes from 587 genes profiled in Bittner et al. (2000), Weeraratna et al. (2002), which were derived based on gene expression data rather than curated regulatory relationships among genes in literature. We believe that the model is appropriate for the purpose of illustrating the effectiveness of objective inferential validity on quantifying the performance of inference procedures in this work. Based on these information, we construct a BNp with the perturbation probability *p* = 0.01. As in the previous studies, the control objective is based on the fact that up-regulation of WNT5A is associated with increased metastasis. Thus, 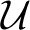 = {**x**|*x*_1_ = 1}. For this network, the undesirable steady-state mass is π_
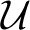
_ = 0.2073 in the original network, which can be reduced as illustrated in Table 3 with different genes as potential targets using the MSSA algorithm on the original network. Based on this model, we simulate 20, 60, and 80 state transitions and infer the network based on these time series data using all five algorithms. As the primary objective here is to reduce the undesirable steady-state mass with WNT5A up-regulated, we focus on its shift derived by the MSSA algorithm based on the inferred networks using different inference algorithms.

**Table 2:** **Regulatory functions in the metastatic melanoma network [Modified from Table 1 in Yousefi and Dougherty (2013)]**.

**Node**	**Gene**	**Boolean function**
*x*_1_	WNT5A	(*x*_3_ ∧ *x*_5_ ∧ ¬*x*_6_) ∨ (¬*x*_5_ ∧ *x*_6_)
*x*_2_	PIR	(¬*x*_1_ ∧ ¬*x*_3_ ∧ *x*_5_) ∨ (*x*_1_ ∧ ¬*x*_3_ ∧ ¬*x*_5_)
*x*_3_	S100P	*x*_7_
*x*_4_	RET1	(¬*x*_1_ ∧ *x*_2_ ∧ *x*_4_) ∨ (¬*x*_2_ ∧ *x*_4_)
*x*_5_	MMP3	(*x*_4_ ∧ *x*_9_) ∨ (¬*x*_9_)
*x*_6_	PLCG1	(¬*x*_4_ ∧ ¬*x*_7_) ∨ (*x*_4_ ∧ *x*_7_ ∧ *x*_10_)
*x*_7_	MART1	*x*_7_
*x*_8_	HADHB	(*x*_1_ ∧ *x*_5_) ∨ (¬*x*_5_ ∧ ¬*x*_9_) ∨ (*x*_1_ ∧ ¬*x*_5_ ∧ *x*_9_)
*x*_9_	SNCA	(¬*x*_1_ ∧ ¬*x*_7_ ∧ ¬*x*_10_) ∨ (*x*_4_ ∧ ¬*x*_7_ ∧ *x*_10_) ∨ *x*_7_
*x*_10_	STC2	¬*x*_3_

**Table 3:** **The shifted undesirable steady-state mass in the metastatic melanoma network by the MSSA algorithm for different control genes derived on inferred networks from five network inference algorithms (REVEAL, BIC, MDL, uMDL, and Best-Fit) with *M* = [20, 60, 80] different number of observed state transitions, compared to the optimal shift by applying the MSSA algorithm to the original network**.

**Control**	**WNT5A**	**PIR**	**S100P**	**RET1**	**MMP3**	**PLCG1**	**MART1**	**HADHB**	**SNCA**	**STC2**
					**OPT (UC)**					
	0.0847	0.1340	0.1767	0.1766	0.1965	0.1965	0.1799	0.0000	0.0259	0.1680
*M*					**REVEAL**					
20	−0.0421	0.1319	0.1702	0.1761	0.1739	−0.1375	0.1777	0.0000	0.0227	0.1027
60	0.0054	0.1316	0.1754	0.0634	0.1961	0.1940	0.1622	0.0000	−0.0448	0.1660
80	0.0728	0.1339	0.1737	0.1727	0.1965	0.1965	0.1795	0.0000	0.0235	0.1678
*M*					**BIC**					
20	−0.0421	0.0789	0.1696	−0.4032	0.1802	−0.1246	0.0026	0.0000	−0.2800	0.0132
60	−0.0421	0.0789	0.1264	0.1765	0.1965	0.1965	0.1799	0.0000	0.0259	0.0023
80	0.0738	0.1340	0.1767	0.1766	0.1965	0.1965	0.1799	0.0000	0.0259	0.1680
*M*					**MDL**					
20	−0.0421	0.0628	0.1696	−0.2655	0.1802	−0.2695	0.0026	0.0000	−0.3586	−0.0574
60	−0.0421	0.0789	0.1264	0.1764	0.1965	0.1965	0.1799	0.0000	0.0259	0.0023
80	0.0738	0.1340	0.1767	0.1766	0.1965	0.1965	0.1799	0.0000	0.0259	0.1680
*M*					**uMDL**					
20	−0.0421	0.0829	−0.2255	−0.2645	−0.2300	−0.1810	−0.2952	0.0000	−0.2295	0.1065
60	−0.0421	0.1309	0.0363	0.1766	0.1965	0.1965	0.1799	0.0000	0.0259	0.0189
80	0.0844	0.1340	0.1767	0.1766	0.1965	0.1965	0.1799	0.0000	0.0259	0.1680
*M*					**Best-Fit**					
20	0.0588	0.1330	0.1724	0.1762	0.1793	−0.0662	0.1796	0.0000	0.0256	0.0115
60	0.0816	0.1340	0.1767	0.1764	0.1965	0.1965	0.1798	0.0000	0.0258	0.1677
80	0.0728	0.1339	0.1737	0.1766	0.1965	0.1965	0.1799	0.0000	0.0259	0.1680

Table 3 compares this network inferential validity μ_ctrl_ for different algorithms. According to the table, even with small sample size, we may obtain effective intervention strategies in most cases from all five inference algorithms. For example, with *M* = 60 samples, RET1, MMP3, PLCG1, and MART1 can be successfully identified as effective intervention targets based on inferred networks using different inference algorithms. These potential targets have been similarly identified in previous publications (Qian and Dougherty, 2008, 2009a; Qian et al., 2009; Yousefi and Dougherty, 2013), which demonstrates the feasibility of deriving effective therapeutic strategies even with partially observed data from the original system. All the algorithms achieve almost optimal performance for all possible control genes when *M* = 80. In fact, Best-Fit appears to obtain the best performance when *M* = 60 compared to all the other algorithms as it better captures network dynamics manifested as steady-state distributions. Hence, Best-Fit appears to be the best-performing inference algorithm when we consider the operational objective to be beneficial alteration of network dynamics. We also note that with small samples (*M* = 20 ), it is relatively difficult to derive effective control based on the inferred network by uMDL; however, when we have enough samples (*M* = 80), we can derive the most effective control for all the target genes based on uMDL. This is again due to its advantage of obtaining consistently close to zero false positive regulators, which leads to the best performance when we have enough samples. This is consistent with the previous results we have seen using simulated networks.

## 5. Concluding remarks

We have considered inferential validity from three perspectives: (1) Hamming distance, which relates to accurate network topology; (2) steady-state distribution, which corresponds to accurate phenotyping because attractors dominate the steady-state mass and attractors correspond to phenotypes; and (3) controllability. From a translational perspective, controllability is an important criterion because a key interest in translational genomics is to derive intervention strategies from gene network models. We have observed from the experiments that controllability provides quite a different view of validation than either Hamming distance or steady-state mass, with performance comparison depending strongly on the number of observations. The upside is that one can achieve decent control when there is still considerable distance between the original and inferred networks relative to Hamming distance and steady-state mass. This depends on network size, connectivity, sample size, and the inference procedure. The general point is that it may be wise to use objective-based measures of validity for practical applications. While the individual components and connections in a system may overall be fairly inaccurate, it may be that those that matter for the objective are determined fairly accurately so that the inaccuracy of the others is of little consequence. The situation is analogous to uncertainty in model classes. While entropy provides an overall measure of model uncertainty, it may be better to use a measure of uncertainty that accounts for the cost of the uncertainty relative to a particular objective because uncertainty that does not negatively impact attainment of the objective is of no practical consequence (Yoon et al., 2013).

### Conflict of interest statement

The authors declare that the research was conducted in the absence of any commercial or financial relationships that could be construed as a potential conflict of interest.

## Figures and Tables

**Figure A1:**
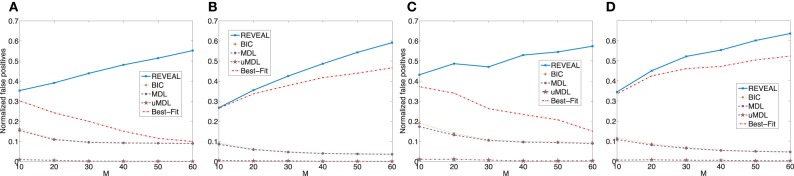
**Comparison of five network inference algorithms by normalized false positive rates. (A)** BNps with 7 genes and *K* = 3; **(B)** BNps with 7 genes and *K* = 5; **(C)** BNps with 9 genes and *K* = 3; **(D)** BNps with 9 genes and *K* = 5.

## Appendix

We plot the normalized false positive rates (the ratio of the number of false positive regulators over the total number of edges) in Figure A1, in which we can see that the performance of different algorithms are consistent as we discussed previously.
